# Neural Distribution–Guided Botulinum Toxin Injection for Platysma muscle: A Split‐Neck Comparison With Conventional Technique

**DOI:** 10.1111/jocd.70645

**Published:** 2026-01-09

**Authors:** Kyu‐Ho Yi, Isaac Kai Jie Wong, Irwan Junawanto, Gi‐Woong Hong, Han Earl Lee, Jovian Wan

**Affiliations:** ^1^ Division in Anatomy and Developmental Biology, Department of Oral Biology, Human Identification Research Institute, BK21 FOUR Project Yonsei University College of Dentistry Seoul Korea; ^2^ You and I Clinic Seoul Korea; ^3^ The Artisan Clinic Private Limited Singapore Singapore; ^4^ Private Practice Jakarta Indonesia; ^5^ Samskin Plastic Surgery Clinic Seoul Korea; ^6^ Opening Plastic Surgery Clinic Seoul Korea; ^7^ Medical Research Inc. Wonju Korea

**Keywords:** botulinum toxins, injections, intramuscular, neck muscles/innervation, platysma, rejuvenation, type a

## Abstract

**Background:**

The platysma muscle plays a pivotal role in the formation of vertical neck bands and contributes to lower facial descent, making it a prime target for botulinum toxin type A (BoNT‐A) in aesthetic neck rejuvenation. Conventional protocols typically involve injecting across the entire muscle, necessitating a high number of injection points and larger total doses, which may increase the risk of bruising, patient discomfort, and potential immunogenicity. Recent anatomical studies using Sihler's staining have demonstrated that motor innervation is predominantly concentrated in the upper portion of the platysma.

**Aims:**

To evaluate a neural distribution–based BoNT‐A injection strategy targeting only the motor‐rich upper platysma, compared with the conventional whole‐muscle injection approach.

**Methods:**

Fifteen patients with prominent platysmal bands received BoNT‐A injections (JETEMA THE TOXIN, JETEMA Inc., Korea) in a split‐side design: The right platysma was injected using a conventional 30‐point technique, and the left platysma received 15 targeted injections in the upper portion based on mapped motor entry points. Efficacy was assessed by the degree of platysmal band relaxation at follow‐up.

**Results:**

Both techniques achieved comparable improvement in platysmal band appearance, despite the targeted side requiring 50% fewer injection points.

**Conclusions:**

Motor innervation–guided BoNT‐A injections may achieve equivalent clinical outcomes while reducing injection burden, toxin dose, and complication risk.

## 1 Introduction

**FIGURE 1:**
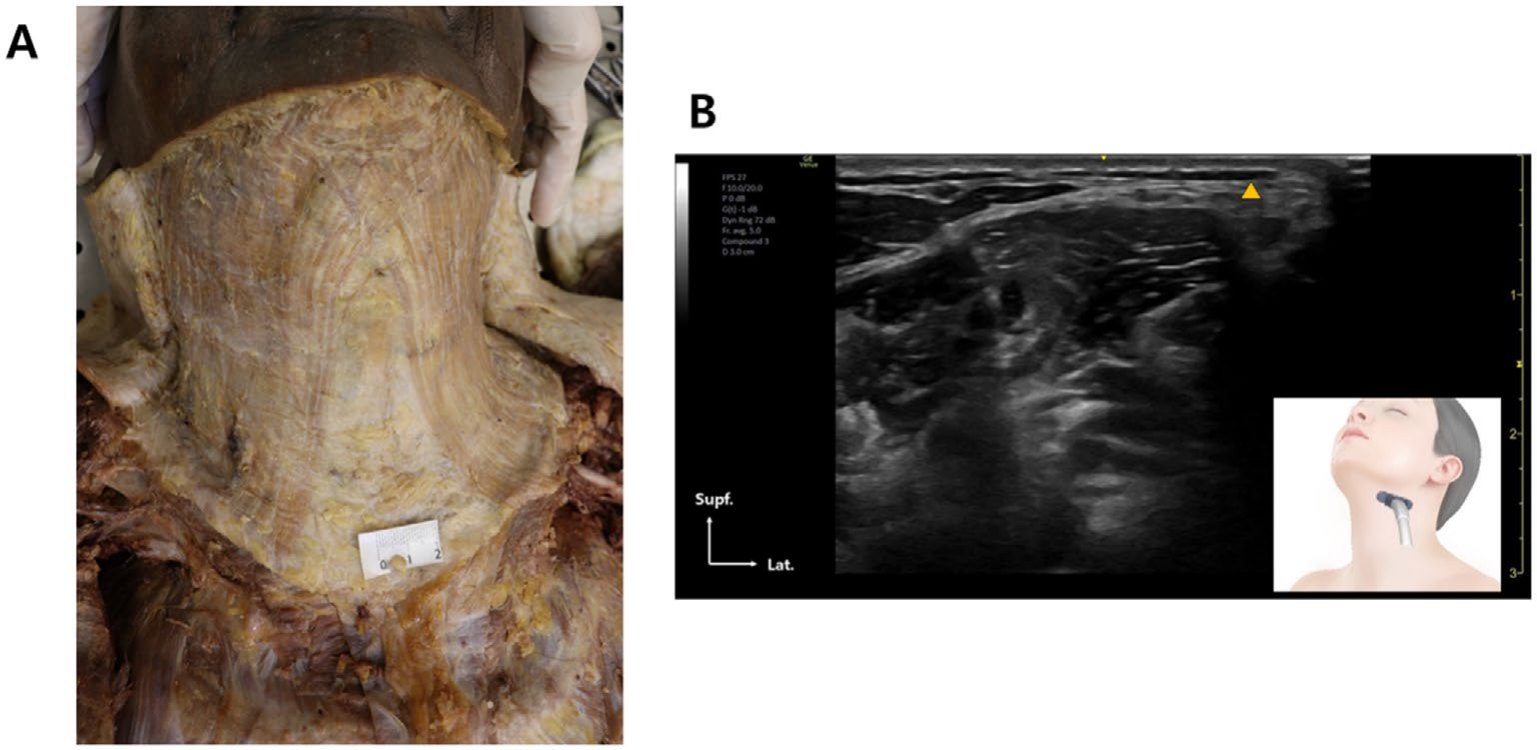
(A) Dissected specimen demonstrating the platysma muscle in situ. (B) Ultrasonographic image showing the platysma muscle embedded within the preplatysmal fatty tissue.

The platysma muscle, a thin, superficial sheet extending from the lower face to the upper chest, plays a central role in the formation of vertical neck bands and contributes to lower facial descent through its depressor function (Figure 1) [1]. These age‐related changes not only affect neck aesthetics but also contribute to loss of jawline definition, making the platysma a common target in nonsurgical rejuvenation procedures. Botulinum toxin type A (BoNT‐A) has been widely used to relax the platysma, thereby softening dynamic neck bands and improving cervicomental contour.

Conventional injection protocols typically involve multiple injection points across the entire platysma muscle, often requiring higher total doses to achieve uniform relaxation. While these methods are effective, they carry several drawbacks, including increased risk of bruising, patient discomfort, higher procedural cost, and the potential for immunogenicity from repeated intradermal or subdermal injections into immune cell–rich tissue.

**FIGURE 2:**
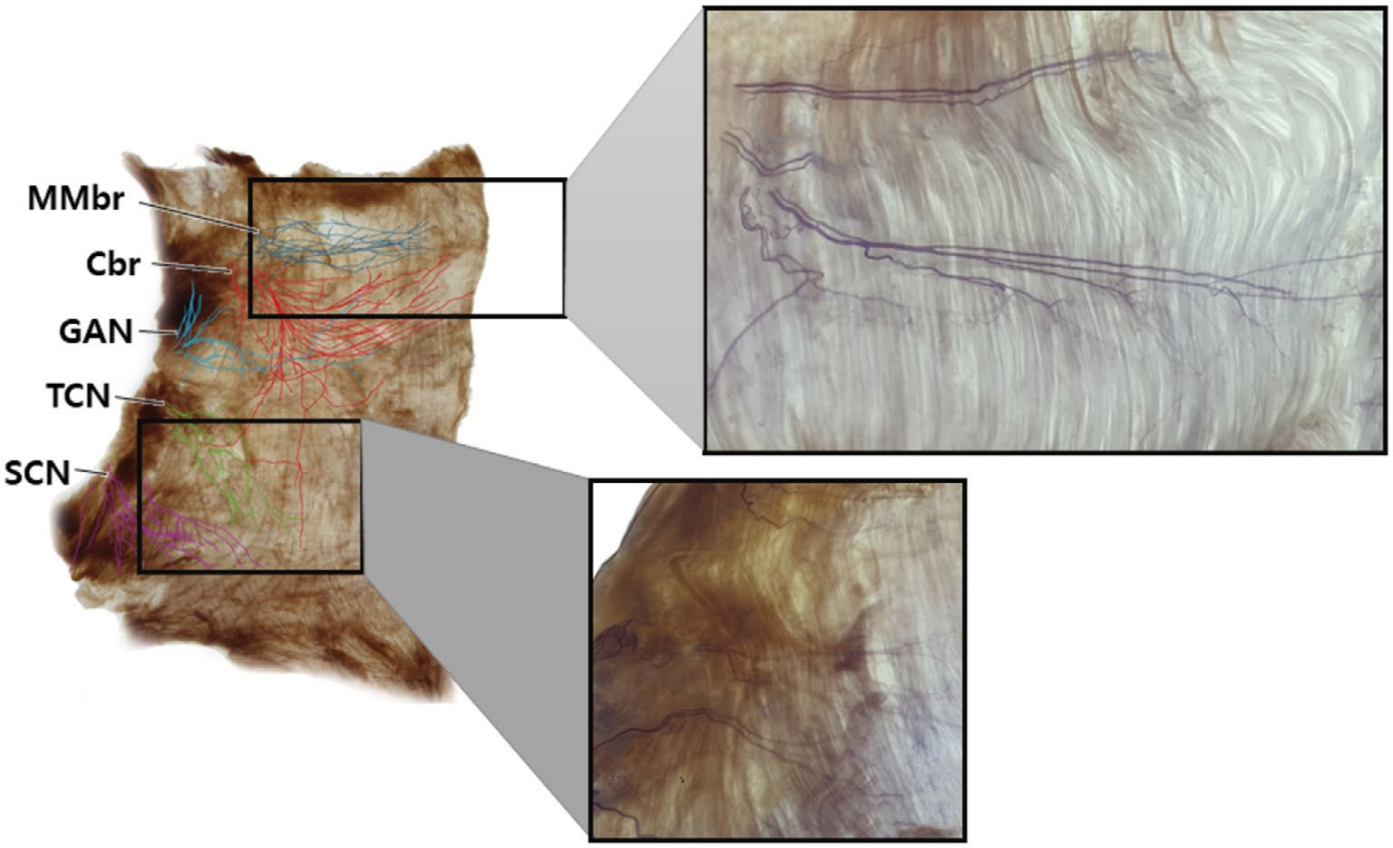
Sihler‐stained specimen of the platysma muscle illustrating neural distribution. The upper portion is predominantly innervated by the cervical branch (Cbr) and marginal mandibular branch (MMbr) of the facial nerve, providing motor control to this region. The lower third is mainly supplied by sensory nerves, including the transverse cervical nerve (TCN), great auricular nerve (GAN), and supraclavicular nerve (SCN), which contribute minimally to muscle contraction, making this region less responsive to botulinum toxin injection for dynamic neck bands.

Recent anatomical investigations using Sihler's staining have revealed that motor innervation of the platysma—supplied by the cervical branch and, near the mandibular border, the marginal mandibular branch of the facial nerve—is concentrated in the upper half of the muscle (Figure 2) [2]. The lower third, in contrast, is primarily innervated by sensory nerves, contributing little to active contraction. This suggests that injections limited to the motor‐rich upper platysma may achieve equivalent functional and aesthetic outcomes with fewer injection points and lower total toxin doses [3, 4, 5, 6, 7, 8, 9].

The aim of this split‐side comparative study was to evaluate the clinical efficacy of a neural distribution–guided BoNT‐A injection protocol, targeting only the upper half of the platysma, compared with the conventional full‐muscle injection approach, using a standardized assessment of platysmal band improvement.

## 2 Materials and Methods

### 2.1 Study Design and Patient Selection

This was a prospective, split‐side comparative clinical study conducted in 15 adult patients (12 females, 3 males; mean age 46.8 ± 7.3 years) presenting with moderate‐to‐severe vertical platysmal bands. Eligibility criteria included:

1. Age ≥ 30 years;
2. Visible dynamic platysmal bands on voluntary contraction;
3. No botulinum toxin injection to the neck within the previous 6 months. Exclusion criteria were neuromuscular disorders, pregnancy, lactation, hypersensitivity to botulinum toxin, active neck infection, or prior neck surgery. All participants provided written informed consent before enrollment.

### 2.2 Anatomical Rationale

Injection mapping was based on Sihler's staining studies, demonstrating that motor innervation from the cervical and marginal mandibular branches of the facial nerve is concentrated in the upper half of the platysma, whereas the lower third is predominantly supplied by sensory nerves.

### 2.3 Injection Protocol

**FIGURE 3:**
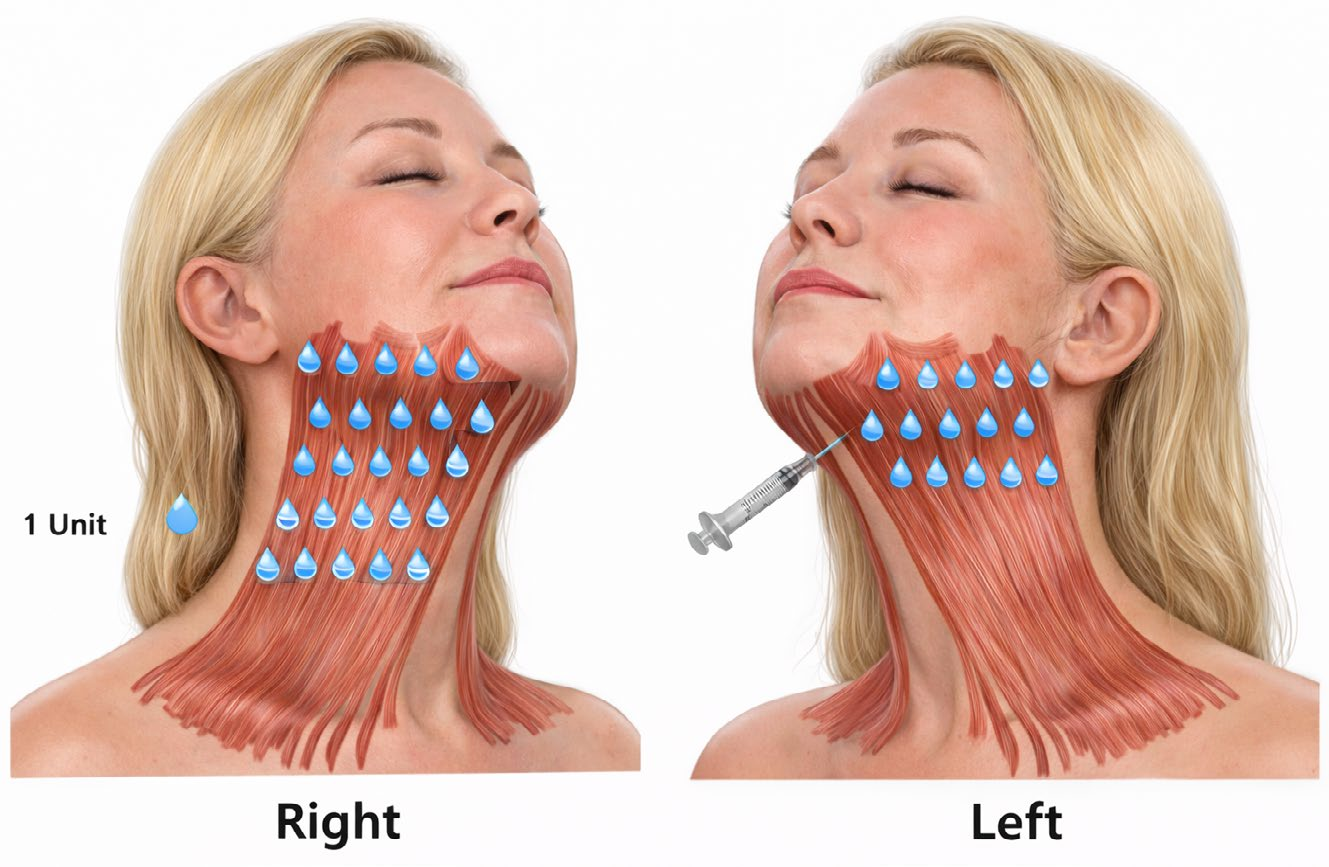
Anatomical distribution of injection points in the platysma muscle and marginal mandibular branch area. Right side (targeted group): 15 points in total—five along the mandibular body, five at the mandibular border, and five located 1.5–2.0 cm below the mandibular border. Left side (conventional group): Same 15 points as the targeted group, plus an additional 15 points placed in the lower portion of the platysma above the clavicular line, totaling 30 points.

BotulinumtoxinA (100 U vial, JETEMA THE TOXIN, JETEMA Inc., Korea) was reconstituted with 5 mL of preservative‐free saline (2 U/0.1 mL). Injections were performed with 1 U per point using a 30‐gauge needle:

- Right side (Conventional group): 30 evenly spaced injection points covering the entire platysma (total 30 U).
- Left side (Targeted group): 15 injection points confined to the motor‐rich upper half of the platysma, spaced 2–3 cm apart along visible vertical bands (total 15 U) (Figure 3).

### 2.4 Outcome Evaluation

At 2 weeks post‐treatment, improvement was evaluated for each side separately by two independent, blinded physicians and by patients using the Global Aesthetic Improvement Scale (GAIS) (3 = very much improved, 2 = much improved, 1 = improved, 0 = no change, −1 = worse). Clinicians also used a 4‐point platysmal band improvement scale (0–3). Both physicians reached complete agreement on the evenness of improvement between sides. Adverse events were recorded throughout follow‐up.

## 3 Results

All 15 enrolled patients completed the study without loss to follow‐up. No serious adverse events were reported. Mild, transient bruising occurred in 3 patients (20%) and resolved within 5 days.

At 2 weeks post‐treatment, both injection techniques produced comparable improvements in platysmal band appearance. Clinician‐assessed platysmal band improvement scores (0–3 scale) were identical between sides (right: 2.2 ± 0.4; left: 2.2 ± 0.4; *p* = 0.92). GAIS scores from clinicians also showed no statistically significant difference (right: 2.2 ± 0.3; left: 2.2 ± 0.3; *p* = 0.88) (Table 1).

**TABLE 1:** Comparative clinical outcomes for right (Conventional) and left (Targeted) platysma.

Outcome Measure	Right Side (30 points)	Left Side (15 points)	*p*
Clinician Improvement Score (0–3)	2.2 ± 0.4	2.2 ± 0.4	0.92
Clinician GAIS (−1 to 3)	2.2 ± 0.3	2.2 ± 0.3	0.88
Patient GAIS (−1 to 3)	2.3 ± 0.5	2.3 ± 0.5	0.94

**FIGURE 4:**
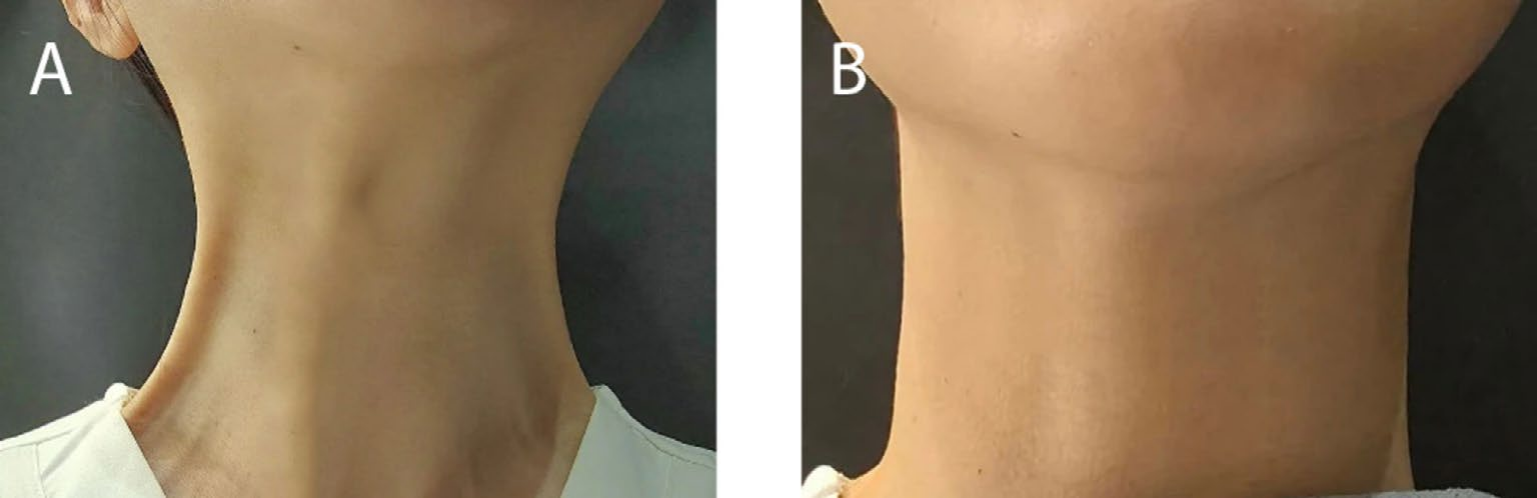
A 37‐year‐old female patient with prominent platysmal bands before (A) and 3 months after (B) BoNT‐A injection. Both sides showed marked improvement, with no difference in Global Aesthetic Improvement Scale (GAIS) scores between the targeted and conventional injection techniques.

**FIGURE 5:**
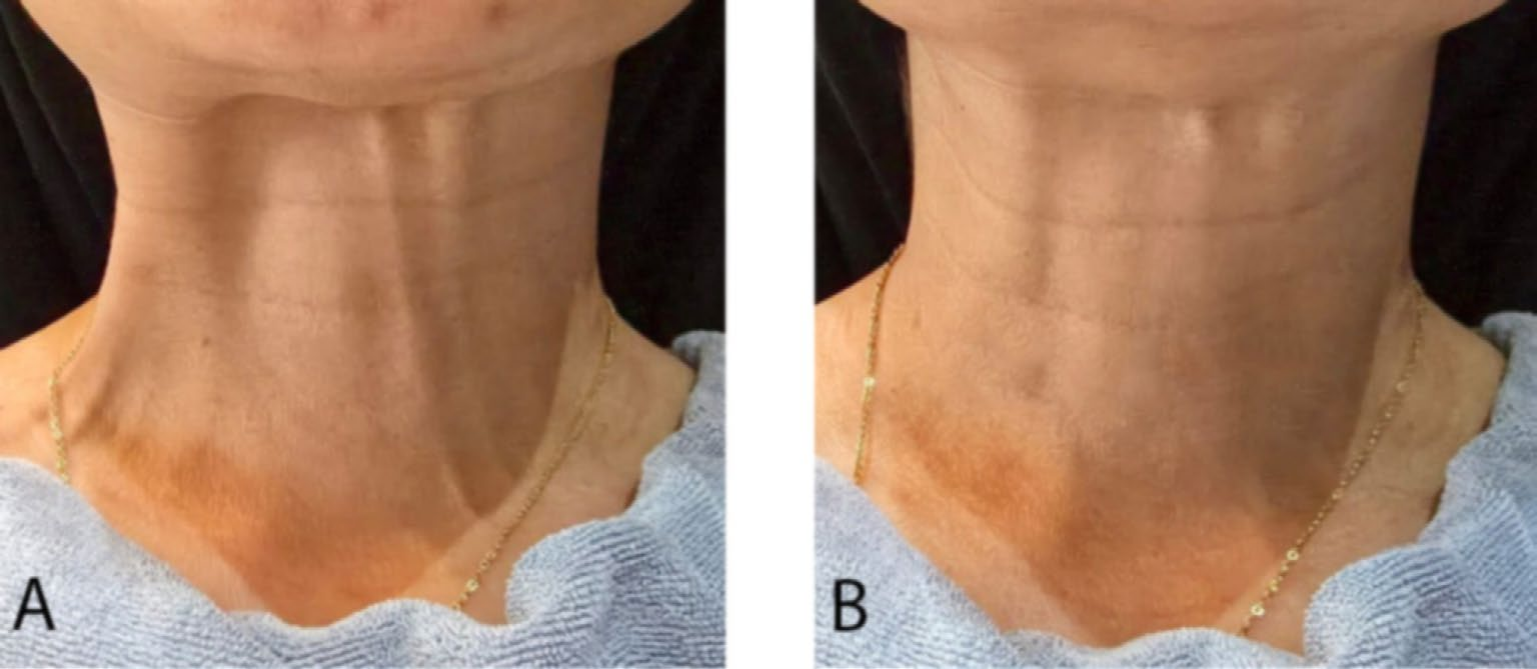
A 53‐year‐old female patient with prominent platysmal bands before (A) and 3 months after (B) BoNT‐A injection. Both sides demonstrated significant improvement, and GAIS scores were equivalent between the targeted and conventional injection techniques.

**Patient self‐assessed GAIS scores** confirmed these findings, with equivalent ratings for both sides (right: 2.3 ± 0.5; left: 2.3 ± 0.5; *p* = 0.94) (Figures 4 and 5).

Two independent, blinded physicians reached full agreement that the degree of improvement was equal between the targeted and conventional injection sides in all patients.

## 4 Discussion

The present split‐side comparative study demonstrates that a neural distribution–guided botulinum toxin type A (BoNT‐A) injection strategy—targeting only the motor‐rich upper half of the platysma—can produce clinical results equivalent to those achieved by the conventional full‐muscle injection technique, despite using half the number of injection points and a 50% lower total dose. This finding supports the concept that injection strategies informed by detailed anatomical understanding can improve treatment efficiency, reduce procedural burden, and potentially enhance patient safety in aesthetic neck rejuvenation.

The platysma is a thin, superficial, and broad muscle extending from the lower face to the upper chest, contributing to vertical neck band formation through repetitive contraction. Its motor innervation is primarily supplied by the cervical branch of the facial nerve, which distributes motor fibers to the upper half of the muscle. Supplementary motor innervation to the uppermost region near the mandibular border is provided by the marginal mandibular branch of the facial nerve. In contrast, the lower third of the platysma is largely innervated by sensory nerves—including the transverse cervical nerve (TCN), great auricular nerve (GAN), and supraclavicular nerve (SCN)—which play minimal roles in active muscle contraction.

This distinct innervation pattern has important clinical implications. BoNT‐A injections placed in the motor‐rich upper half of the platysma are most effective in achieving muscle relaxation and reducing the appearance of dynamic neck bands. Conversely, injections in the lower platysma, dominated by sensory innervation, are unlikely to yield substantial therapeutic benefit and may increase the risk of adverse effects, including weakness of neck support structures and functional compromise. Furthermore, injection into the lower neck musculature may inadvertently affect deeper cervical muscles, particularly if the toxin is delivered below the platysma, increasing the risk of dysphagia.

Based on the neural distribution, injections should be concentrated in the upper half of the platysma along visible vertical bands formed during contraction. These points are generally placed 2–3 cm apart, with 2–3 points per vertical band. In cases where banding extends to the mandibular border, additional injections—typically 1–2 per side—should be placed in the marginal mandibular and cervical branch innervation zones to soften the neck–jaw transition and improve contour.

Ultrasound guidance offers additional benefits in accuracy and safety by ensuring the needle or cannula is placed in the subdermal or subplatysmal plane to effectively target muscle fibers while avoiding intradermal deposition, which may increase the risk of bruising, immune sensitization, and reduced efficacy over time (Figure 1). Depth should be tailored to patient BMI; individuals with thicker preplatysmal fat may require deeper placement to ensure delivery into the muscle, while injections below the muscle should be avoided to prevent weakening of deeper cervical musculature [7, 10].

This targeted approach reduces the number of injections and the total BoNT‐A dose, resulting in less discomfort, shorter procedure time, lower likelihood of bruising, and decreased treatment cost—important considerations in high‐volume aesthetic practices. Additionally, limiting injections in immune cell–dense dermal and subdermal layers may reduce the potential for neutralizing antibody formation, preserving long‐term responsiveness to BoNT‐A. From a functional standpoint, sparing the lower platysma helps maintain neck stability, which is particularly beneficial in older patients or those with pre‐existing cervical weakness.

Our results are consistent with prior anatomical studies and clinical trials, suggesting that treatment efficacy is highest when injections are localized to motor innervation zones. Prager et al. demonstrated that targeted injection in platysmal bands achieved satisfactory aesthetic outcomes without full‐coverage injections, and Sugrue et al. emphasized the importance of anatomical precision to minimize side effects. Similarly, recent Sihler's staining–based analyses have confirmed that the motor endplate regions in the platysma are concentrated in its superior half, providing a strong scientific basis for limiting injections to this region.

Despite these promising findings, several limitations must be acknowledged. The study involved a relatively small sample size (*n* = 15) and short follow‐up (2 weeks), which may limit the generalizability of the results. The observational design lacked randomization and objective quantification of muscle activity (e.g., via electromyography). The outcomes were based on clinician and patient‐reported scales, which, while clinically relevant, are inherently subjective. In addition, this study did not assess the long‐term duration of effect, which is essential to confirm whether dose reduction impacts the longevity of results.

Future research should involve randomized controlled trials with larger, more diverse patient populations and extended follow‐up periods. The incorporation of objective assessment tools, such as high‐resolution ultrasound imaging, three‐dimensional photographic analysis, or electromyography, would allow for more precise quantification of muscle relaxation and aesthetic improvement. It would also be beneficial to investigate the durability of results, cost‐effectiveness, and patient satisfaction over multiple treatment cycles to determine the long‐term sustainability of this approach.

## 5 Conclusion

This split‐side comparative study demonstrates that a neural distribution–guided botulinum toxin type A injection protocol, targeting only the motor‐rich upper half of the platysma with 15 injection points, achieves equivalent aesthetic improvement to the conventional 30‐point full‐muscle injection. By reducing total dose and injection burden without compromising outcomes, this technique offers a more efficient, cost‐effective, and potentially safer approach to platysmal band treatment. Incorporating anatomical knowledge of motor innervation can refine clinical practice, minimize patient discomfort, and reduce the risk of immunogenicity, supporting its adoption as a preferred strategy for neck rejuvenation.

## Author Contributions

Conceptualization, Kyu‐Ho Yi, Jovian Wan, Han Earl Lee. Writing – Original Draft Preparation, Kyu‐Ho Yi, Jovian Wan, Irwan Junawanto; Gi‐Woong YU; Han Earl Lee. Writing – Review and Editing, Kyu‐Ho Yi, Jovian Wan, Isaac Kai Jie Wong. Visualization, Kyu‐Ho Yi, Jovian Wan. Supervision, Kyu‐Ho Yi. All authors have reviewed and approved the article for submission.

## Funding

The authors have nothing to report.

## Ethics Statement

This study was conducted in compliance with the ethical principles outlined in the Declaration of Helsinki.

## Consent

Informed consent was obtained from all participants, with full disclosure of the study's purpose, risks, and confidentiality.

## Conflicts of Interest

The authors declare no conflicts of interest.

## Data Availability

The data that support the findings of this study are available from the corresponding author upon reasonable request.
